# The Centralization of Energy

**Published:** 1882-11

**Authors:** Edwin Wooton

**Affiliations:** Hon. Lecturer on Physiology and Psychology to the Society of Science, Letters, and Art, London


### THE DENTAL REGISTER.

VOL. XXXVI.] NOVEMBER, 1882. [No. 11.

### COMMUNICATIONS.

### The Centralization of Energy.

By EDWIN WOOTON, Hon. Lecturer on Physiology and Psychology to the Society of Science, Letters, and Art, London.

No man can judge accurately of the consciousness of beings other than himself, save from external phenomena. In saying this, I do not mean to impeach that department of psychology known as "mental communion," for this latter condition is abnormal, and I am here dealing with the common life. Language, in the ordinary sense of the word, is a gift enjoyed by no earthly being besides man, yet the lower members of the animal world exhibit phenomena which man ever recognizes as indicative of their mental state. The reason of such recognition is very simple —immediately we advance our inquiries beyond our own kind, we can only judge of mental states from analogy; nay, we are restricted to this when we are dealing with those of our own species who are dumb and possess no means of verbal communication.

Even in his highest condition man is constantly showing by outward signs the thought or impression of the mind, and being aware of the limited faculties of the brute, he confines his expectation to its known ability of expression; in other words, if the actions which in man are associated with some particular emotion or sensation are performed by the brute, so far as its ability permits, man attributes to the inferior animal the same mental state.

Nevertheless, the same analogical reasoning of many becomes narrow, and of these some lay it down as an axiom that no animal in whom a distinct cephalic ganglion has not been developed can possibly *appreciate* impressions; others, not embracing the above view, yet hold that where a cephalic ganglion exists it is the sole seat of volition and sensation, and assert that deprivation of such ganglion leaves the animal either lifeless or a mere automaton—in any case, a non-sentient, non-volitional piece of organic matter.

Two questions then arise for our consideration:

First—Are volition and sentience faculties common to the animal kingdom?

And next—What organs are necessary for their manifestation?

The importance to biological science of a correct solution of these problems it is difficult to overrate. In animal physiology—every fact discovered, every law demonstrated, every secret wrung from the bosom of nature is a stone cut and smoothed, and which will find its place and duty in the great fabric which science is building in the very presence of, and as a defiance to, the assaults of ignorance and disease.

### THE CENTRALIZATION OF ENERGY.

All bodies—animal and vegetable—are composed of a substance termed Protoplasm; this is a chemical compound represented typically by the following analysis:

| | |
|---|---|
| C | 53·5 |
| H | 7·0 |
| N | 15·5 |
| O | 22·0 |
| S | 1·6 |
| P | 0·4 |

Physically the basis of life varies from semi-viscidity to the hardness of ivory; the former is its simplest state, and that which is always found in the least organized animals, the latter condition and all intermediate states, are produced by the addition to the protoplasm of various chemical substances. Protoplasm exists in the form of minute circumscribed masses, to which the term "cell" is applied. With our present knowledge the least differential beings which can with certainty be assigned to the animal kingdom are the Monera. These are minute marine animals utterly destitute of organs: their bodies are not homogeneous, but consist of two parts--first, an outer, more dense and apparently structureless portion, which answers to a membrane, covering, or cyst; and, secondly, the greater part of the body substance which is granular, more fluid and mobile.

It is in the Gregarinidæ that we first see an alteration in the animal structure. An adult Gregarinidæ differs structurally from a Monerum in this respect only—there is in one portion of the body of the former a harder particle, termed the nucleus, enclosing another, the nucleolus; of the purpose of these parts we shall speak by-and-by.

In the Amoepæ there is added a contractile space, concerned in the circulation.

The Foraminifera possess an external covering or shell.

The ciliated Infusoria have a permanent oral aperture and digestive cavity, and the exterior of the body has at one or more parts vibratile appendages termed cilia.

Rudimentary eyes make their first appearance around the circumference of the necto-calyx in the Discophora. Rudimentary organs of hearing are found in the same animals and in the same region. The eyes, or "ocelli," are segmented cells; the auditory organs, or "vesicles," are rounded sacs lined by epithelium, and containing one or more solid motionless concretions composed of carbonate of lime, immersed in a clear fluid.

A differentiated nervous apparatus first shows itself in the Ascidian Mollusc. It consists of one ganglion situated in the neighborhood of the mouth in the mantle, and giving off cords which proceed to the sense and digestive organs, the muscular sac and both orifices Next above the Ascidians we may place the Ctenophora, in which, within the apical pole, that is the end farthest removed from the mouth, is the Ctenocyst, a spherical vesicle; this, which is a sense organ, rests on a small ganglion giving off fibers. In the Actinidæ there is a system of branches supplying the ocelli, at the bases of the tentacles, and also the muscular tissue. Mingled with these fibers are cells or ganglia. The next well marked stage of development may be seen in the Echinoidea. There is a ganglionated cord surrounding the gullet, and sending off five branches among the ambulacral spaces. The

Annulosa have a chain of ganglia running the length of the body and united by nervous cords. At one extremity, where the sense organs are situated, the last post-œsophageal ganglion gives off two cords; these pass one on either side of the œsophagus, and enter each a pre-œsophageal ganglion--the cephalic—this ganglion is generally double. This description applies to all annulose animals, but the highest members of the order—the Insecta, have two cords passing backwards from the cephalic above the ventral ganglia, and giving off branches to them and the body walls, &c.

This is the most rudimentary form of the cerebro-spinal system.

The Vetebrata are animals in whom a vertebral column is present. Such column may persist in the rudimentary form of the notochord, or may be developed into a bony axis composed of distinct segments. The body consists of two tubes; the greater, the anterior—containing the viscera of digestion, excretion, and circulation; and a lesser, the posterior, containing a nervous cord which anteriorly is connected by nerves to organs of sense. The vertebrate with the simplest nervous system is the lancelet. The neural axis of this animal is a delicate tract of nucleated cells surrounded by a coating of pia matter. It tapers to both ends, more however, towards the posterior. Fifty or sixty pairs of nerves are given off laterally. In the diodon the cerebro-spinal column has its anterior extremity enlarged into a distinct cephalic mass, behind this there is a portion of the column, from four to twelve inches in length; this shows transverse white striæ and contains gray matter; it is a nervous center and a portion of the brain. The rest of the neural canal is occupied by a "cauda equina." The lampreys and hag-fishes are of a higher nervous organization than the above. They have a cartilaginous cranium, and the spinal cord extends slightly anteriorly; but there is no bony spinal column, and the notochord is persistent. The cord in the diodon is divided by shallow anterior, posterior, and lateral fissures. The posterior fissure at the cephalic extremity is deepened, and the halves diverging expose grey matter.

In the cod and the shark the posterior fissure widens and the halves of the cod expand; the nerve tissue bounding the myelonal canal becomes swollen and rounded, and the columns diverging as they advance, expose an intermediate nodular tract. Two lateral "vagal" columns also project into the ventricle from the conjoined restiform and posterior pyramidal tracts.

The next structures added are a cerebellum and crura cerebelli.

The primary division of the brain may be said to consist of a medulla, a cerebellum, and one or two appendages of no great importance. Relatively it is both larger and more complex in fishes than in the higher vertebrates. The brain is essentially the developed cephalic portion of the cord.

The second division consists of the "optic lobes." This is generally the largest division in osseous fishes. Beneath the "lobes" are two subspherical bodies, "hyproaria," separated by walls containing a cavity, which is the homologue of the third ventricle in man. This ventricle is prolonged downwards into the pedicle of the pituitary gland and upward into that of the pineal gland.

From the third ventricle fasciculi we continued forward to the anterior aspect of the optic lobes, where they form two small masses of gray matter; these are rudimentary cerebra.

While the brain of the crocodile is scarcely larger than the thumb of an adult human being, that of the bird has expanded both laterally and vertically, but is still composed chiefly of the optic lobes and the cerebellum.

In the dog and in other animals the brain has advanced more anteriorly, and the cerebra have coincidently developed. The degree of such advancement and development being of course collateral with the animal's degree of intelligence. This rule holds sway even amongst the various human races.

It can with certainty be demonstrated that the porosity is a property or condition of all matter; contact is never absolute, but always relative. The composite-particles of the protoplasmic cell are, therefore, not in coaptation, but separated by infinitesimal pores or spaces. The passage of material in a state of fluidity through such spaces into the cell substance is termed intussusception. The cell ordinarily maintains its existence by means of this process. Under certain circumstances, however, the protoplasmic cell, if capable of extension, as in the amœbæ, will flow round and encompass a solid particle of nearly its own diameter; it will then accomplish the perfect digestion and assimilation of its prey. The modification of protoplasm is termed differentiation; the latter is both structural and functional. As I have said, the simplest cell presents merely a slightly condensed margin and a granular homogeneous body substance. But as the pedigree of the free cells lengthens, canular spaces become marked out; the protoplasm bordering these channels assumes a more active part; there is an increase of energy in certain paths for the benefit of the whole body. Thus, then, the simplest structural differentiation is the relative consistency of the ectoplasm and endoplasm, the simplest functional differentiation is their relative activity.

The functions of the nucleus and nucleolus are sufficiently evident; by their segmentation they commence the process of proliferation by cell division.

The contractile vesicle of the amœba is the simplest form of a circulatory apparatus to be found in the animal kingdom; the fluid which it propels is the food of the animal derived from the medium in which it is living.

The differentiation of function we have been hitherto considering is merely the increased localization of contractility.

The latter condition might at first sight appear to be exemplified on the vibratile cilia of the monera, but this is not really the case. The cilia of these animals are no more vital in function than the remainder of the protoplasm, for they are retraced and fresh portions of the body substance protruded. Their movements are merely due to their relative length and fineness. In a similar manner the emission of amœboid pseudopodia can be regarded only as a special manifestation of the vitality of the whole protoplasm.

There is, and must be, in every animal the action of individual parts for the benefit of the whole. What, then, is the difference between the simple protoplasmic cell and a body possessing the nervous-muscular and other tissues?

The answer to this will appear as we proceed. Ordinary protoplasm exhibits a gradually increasing localization of energy or function in certain parts and cells, producing a progressive differentiation of structure to perfect nerve and other tissues.

Differentiation of structure then is the sequence and accompaniment of differentiation of function, and is produced by persistent maintenance, with gradual increase of function.

Objects external to any being possess the properties of form, extent, specific gravity, density, temperature, color, and chemical constitution. They have position and may give rise to ærial vibrations.

Position is the one and only property of all matter. Size, form, specific gravity, density, color, temperature, and *vitality* are merely due to the arrangement of the constituent parts.

Temperature is the result of the constant positing of material to a greater or less degree; the positing varying as the heat. Chemical combinations are, of course, the result of position. Chemical elements are probably but one form of matter, having their infinitesimal particles differently arranged. These properties then constitute an *objective* differentiation, and if there be a *subjective* differentiation of individual beings, it must be correlated to the former. The sense faculty in man is separated into five divisions—sight, hearing, touch, taste, smell. The organ of sight can appreciate size, apparent density and color. That of hearing can appreciate sound only. The organs of touch estimate size, density, and temperature, and this last whether the skin come into contact with the object, or the sensation be communicated by a medium. Smell and taste distinguish empirically certain chemical states. The latter being constant, the sensation excited is also constant. Approval or dislike is merely a judgment on the merits of the sensation.

The objective qualities of matter, its objective differentiation, are always present. If we select a particle 100,000th in. in diameter, we have a circumscribed portion of matter possessing form, gravity, density, color, etc. *We* could not see it if we hadn't it under the microscope; but it is evident that the simple free cell is possessed of a capability of appreciating the presence of minute atoms, for the said cell captures and digests the particle in question.

Probably the simplest sensation is touch; undoubtedly, as shown above, the cell possesses this. Yet this sense must differ in kind from our own. Contact must, in the microscopic animal of which I am speaking, excite a sensation of chemical combination or condition, interpreted by the being as edibility, for the cell will reject unsuitable food.

The qualities then required for a living sentient being of the cell's anatomy, would be the capability of appreciating contact with substances edible and inedible; i.e. with beneficial and injurious objects. Now, since the animal does not show his appreciation of objects unless he be in contact with them, we must suppose contact the sole medium of sense. Now contact can only give rise to an idea of the properties of the article in contact, and of the parts in actual apposition. We know also that sight depends on waves of air, and cannot be communicated by contact of a tissue with a substance. Sight may be a variety of touch, but we have no reason to suppose that anything analogous to the sensation can be excited by direct contact. Considering, then, the *objective* properties of the particles constituting the cell's food, we may form a good notion of the *subjective* properties of the cell. Thus, then, the subjective is correlated to the objective.

We shall see that as the body becomes by hereditary education more capable of appreciating the properties of matter, separate cells become appropriated to the consideration of one or more of these properties. The action itself modifies the tissue into the form most suitable for it, and this by use is elaborated, and by heritage perpetuated. Differentiation of sense is correlative with differentiation of tissue. The former being the starting point: life is the cause of organization.

The nervous, muscular, and other systems are merely the total assumption in an animal by certain cells of the common properties of protoplasm. In any being, if we perceive phenomena indicating the possession of sense, we must attribute sense organs to that being. The whole body may be a medium of sensation, in which case it is only perceptive by immediate contact. Yet, we are not to assume that such structural differentiation must be visible to the eye. It may be that certain cells or particles in a body may assume any one of the common properties of protoplasm to a greater extent than the remainder, without undergoing any immediate structural differentiation. Neither are we to assume that where an animal can appreciate the presence of an object only by immediate apposition, the tactile sense is so simple as on the epidermal surface of man. On the contrary, we are forced to believe that since the higher tissues of the vertebratæ are merely the elaborated protoplasm, so the differentiated senses of the former are found in the latter, as embryonic undoubtedly, but more complex than epidermic touch. Then, is the protoplasmic cell percipient?

All tissues are composed of this substance in a simple or modified condition. Now, all inquiry tends to show that percipience is not a property of any tissue, but that it is merely a *via animi.* Were, however, this opinion to be proved false it would not affect the question. Percipience is not a visible phenomenon, we judge of its existence in any being other than ourselves merely from analogy. Again, the remaining properties of the tissues of the highest animals, e.g. assimilation, contractility, and excretion, are performed perfectly by the simplest protoplasm. Lastly, the protoplasmic cell is a living individual, we must, therefore, bring the question of its possible percipience to the test of analogy. If one drop of strong hydrochloric acid be added to about twenty of water containing rhizopoda, the latter appear to shrink up, after exhibiting phenomena indicative of violent stimulation, and the pseudopodia are no longer emitted. If now to the liquid be added ammonia, equal in strength and quantity to the acid, so as to neutralize the action of the latter, the rhizopoda lose their contracted appearance and once more emit pseudopodia, although these movements are feebler than in unmixed water, thus showing that the neutral fluid is an unhealthy medium for their existence. Now, in this experiment we have a perfect example of reception of impressions. The cessation of movement is not owing to destruction of life, neither is the shrinking of the animal the mere result of chemical action, for if the experiment be repeated with dead rhizopoda, the latter do not shrink up. Of course, if the strength of the fluid be sufficient in either case, a process of cauterization ensues, and the rhizopoda disappear.

We conclude, therefore, that the protoplasmic cell is percipient, and that the tissues of the highest animals contain no new property, but simply the elaborated and concentrated qualities of simple protoplasm.

In the humblest cells, each has to digest its own food. When several unite to form a being, certain of them are set apart to form the boundaries of a cavity and to fulfill the office of digestion for the benefit of the whole body; the other cells or particles do not digest. There is a cessation of function in parts, and an increase in others. In the same manner one or more of the other faculties of protoplasm is assumed by separate cells, excepting, of course, assimilation, which is the common property of tissues. As these faculties become separated the structure of the cells differentiates, special functions producing and being assumed by altered structures.

Protoplasm, then, is capable of all the vital functions without an absolute differentiation into separate tissues.

I have said that the simplest sensation is the tactile. The five senses of man are differentiated forms of touch. The difference in the sensations corresponds to the variations in the stimuli which are capable of affecting the several organs. Now "touch" is the power of appreciating contact, and such contact may be ærial or direct.

Aerial contact is effected by waves of aether. Such waves are continuous or vibratile.

Continuous waves in impinging on a sensitive apparatus give rise to the sensation of the totality of the waves, or light. Vibratile waves are due to the vibrations of material bodies, or to obstruction to the passage of currents of air. Such waves are interpreted as sound. Currents of air impinging in any vertebrate on any part of the body not set apart for the appreciation of vibrations are interpreted merely as contact—the sensation affording no indication of the origin of the waves, or the objects over or through which they may have passed. Ærial motion then conveys to the eye the idea of the presence of objects. Objects have their statical or passive quality, and their active or vibratile quality. Vibrations cannot give rise to an idea of their causes; this is the result of education. Still there is a permanent distinction between vibratile and continuous waves. Direct contact may be effected by the immediate apposition of the object itself or of its gaseous particles with the being. The former is the property of the simplest protoplasm. By inherited education the sense of touch becomes refined; the protoplasm is at length percipient of the contact of *aether*, of the continuous waves into which the vibratile are merged and lost. As the protoplasm become thus sensitive special cells—special points on the outer surface—are more precipient than the rest of the body. Acted on by the aether the molecules are forced into that position which the easier admits of the impression of the external force; hence we obtain a structural differentiation. Such alteration or modification of tissue takes the form of ocelli for the continuous and of auditory vesicles for the vibratile waves. The most refined form of direct tactile sense is the olfactory. Minute particles of the substance impinging on a sensitive apparatus give rise to sensation—which is referred to the properties of the object from whence the gaseous particles were separated.

Taste is not nearly so refined a sense as the foregoing, which, according to Valentin, can discern $\frac{3}{100,000.000}$ of a grain of musk. Nevertheless they are both examples of sensation excited by direct contact, and they are never totally separated, even in the highest vertebrates, although under ordinary circumstances the organ appropriated to each is enabled to act independently. A differentiated apparatus for the appreciation of the qualities of an object, as odor and taste undoubtedly makes its first appearance in the form of antennæ. But the only animals with antennae which in the presence state of science we can certainly declare to be media of taste and smell are the insecta. There can be no doubt that the antennæ of these animals are sense media. The arguments in favor of this view rest on the following considerations. If in any intersect possessing these organs they be cut off close to their bases the animal is unable to discover the neighborhood of strongly odorous saccharine material. The animal appears capable of exercising little or no choice in the selection of its food until the antennæ are renewed. If a common butterfly be watched after it has alighted on a flower, it will be seen that the animal continually points the antennæ at various parts of its banqueting table; depressing them until their apices are in contact with the petals. Moreover, the antennæ are raised and depressed, adducted and abducted, until an ap- parent satisfactory sensation is excited, when the animal follows the direction indicated by the last position of the antennae.

The latter form, indeed, the insect's exploring organs, they are far more important to its existence than an optical apparatus. It is a self-evident fact that the sense of smell among the insecta must be remarkably acute. They can detect saccharine material of whose presence man is utterly unconscious. They will be at- tracted by hundreds to a flower garden in the midst of a sterile country. The one organ is probably capable of transmitting the two sensations of odor and taste. The difference as inferred above rests in the method of using the antennæ. When they are elevated and pointed in any direction smell is exercised; when they are depressed and opposed to any substance the sense of taste. That the insect can detect the neighborhood of its food by the sense of smell to a greater extent than by that of sight is also sufficiently proved by the fact that they will make their way to such food even when it is placed in such a position and under such circumstances as entirely preclude the possibility of its being seen by the animal. Thus the blackbeetle will find its way to the darkened closet, mount the walls, and proceed unerringly until it has arrived at the provisions it is seeking. It is worthy of note that the butterfly is a hater of gloom and a lover of sun- light, it seldom finds its way into any dwelling.

Simple touch or contact is in man a property of the whole ex- ternal integument; in certain parts, as in the palms of the hands and tips of the fingers, it is refined, and the properties of an ob- ject can be much more readily estimated by these parts than the general surface of the body. Indeed, the extremities of the digits are endowed with a sensitiveness which is differentiated from that of the rest of the integument and may be said to the second step in the development of tactile power, which latter pro- ceeds, as we have seen, to sight and hearing. For these reasons, it is extremely difficult to say with certainty what animals possess organs of simple touch or more definitely where the simplest differentiation of tactile function is to be found. However, simple an apparatus may appear, it is yet possible for it to be the means of conveying more *complex* sensations than its analogues in the more highly differentiated animals. Certainly the pseudopodia of the actinophrys sol are organs of touch. Certainly, also, they are tolerably persistent, and entitled, therefore, to be considered structually differentiated. Neither can there be any doubt that they form the simplest example of structural tactile differentiation. The exact estimation of the tactile power of these pseudopodia must, however, remain for obvious reasons an uncertainty. Of one thing we may, however, rest assured; they are not sensitive to ærial, but merely direct contact. Another fact is tolerably plain--they are not excited by such contact in the form of gaseous particles. The fact however, remains that they can distinguish between edible and inedible substances. Unless this be due merely to the consistency of the latter we must suppose their pseudopodia organs of elementary taste as well as touch.

The situation of nerve organs in an animal will materially depend on its fixture or freedom. The spinal nerve organ is but the continued differentiation which is seen in the mouth, etc.

The sense organ will ever be developed on those parts which are most called on for perceptive faculties in order to minister to the welfare of the being. Thus the fixed animal will not require special perceptive faculties at its base. Animals capable of swimming swiftly forwards and of turning quickly, will require only sense organs in the part of the body which lies in the direction of movement. But animals cumbrous in their form, incapable of swift motion, will require organs which shall inform them of the neighborhood of objects in every direction around their bodies. Hence, in the ctenophora we see a mouth with round sensitive lips; at the opposite extremity a distinct sense organ of sight. On the apical side of the equator Ctenophores arise. These are not mere organs of locomotion, they are media of sense protecting the sides of the oral pole.

In considering the physiology of the invertebrate nervous system, we are brought at the outset to the question of the possible conducting power of protoplasm. Organs of sense make their appearance before nervous cords, but all analogy would teach us that sense organs are not themselves perceptive, in other words, that they merely transmit the impressions they receive to the body subtance. Moreover, in an animal possessing ocelli, but no nervous cords, the body acts upon an idea or impression received through these sense organs. Now, the very fact that the animal has the power of controlling the movements of differentiated parts is a sufficient proof that impressions must be conveyed through definite tracts to distinct loci: in other words that conducting power is assumed by cells prior to their structural differentiation. For these reasons we may look on ocelli and kindred organs as the primary alteration of sensitive tissue or the simplest example of the alteration of structure which protoplasm undergoes to meet the requirements of perception and action.

It is not merely probable, but absolutely certain, that the nervous system does not make its first appearance as an acephalic ganglion. It would be very pretty and diagramatic to show that it did, but it does not. After the establishment of an oral aperture and ocelli, a small tract in the neighborhood of the mouth becomes altered in structure, it is a circumscribed rounded mass, from which cords proceed to the sense and digestive organs. This occurs in the Ascidian Mollusc. *Because* this ganglion gives fibers to the sense organs, the only ones indeed that they receive, and that they are not motor organs, it follows that the ganglion must be a center of sensation. But, taking into consideration the small number of fibers distributed generally, it is manifest that the nerves connected with ganglion cannot be the sole conductors of sensation. In other words, the non-nervous mass of the body is sensitive. Irritation of any part of its body causes an immediate contraction of the muscular coat, resulting in the passage of a jet of water from the orifices of the body.

From the preceding considerations I am compelled to conclude, that solitary as the ganglion of the Ascidian is, the energy of the body is not centralized in it. I summarize the functions of the animal as follows:—

The organs of special sense, when present, transmit their impressions to the ganglion.

The ganglion is an elaborating, a perceptive, and a distributing organ.

The whole body is sensitive.

Sensation is more exalted in the ganglion than the remainder of the body.

It is a reflex centre for the digestive system.

The question now arises whether the ganglion is the simplest form of the cephalic ?

It might be thought, perhaps, that the one part of its being directly connected with the organs of sense, sufficiently indicated the relations of the ganglion to the nervous systems of the vertebrates. But if we consider that it is the only nerve centre in the animal, that it directly governs the digestive viscera and the apparatus of motion, that the highest nervous systems have for these and other offices separate and distinct ganglia, we shall rather regard it as an undifferentiated analogue of the whole vertebrate ganglionic apparatus. Just, in fact, as the functions of the man are found in a rudimentary generalised condition in the protoplasmic cell, so the specialization of function has, in the ascidian, marked out a nodule which is the generalized representative of the totality of man's ganglia, each of which has special functions.

Before, however, such specialization occurs, elaboration of the sense media takes place. This occurs in the ctenophora, in whom the ctenocyst is in direct communication with a nerve ganglion. Multiplication of ganglia now sets in and we can select no better example of this than the Actinidae. Such multiplication is accompanied by a corresponding differentiation of ganglionic functions. Some are devoted to the purposes of sight, others to controlling the muscular tissue, etc. It is most important to note that throughout the Protozoa, Coelenterata, Annuloida, and acephalous Molluscs, whatever the arrangements of any nervous or sensitive systems, there is no spot, nodule or ganglion of supreme importance over others in the animal economy. The assidian, I have said, possesses the simplest nervous apparatus, but with the increase in the number of ganglia which occurs in many animals belonging to the above divisions there is no persistent maintenance of a single ganglion connected with organs of sense. Were it so we could only arrive at one conclusion—that a cephalic ganglion was the first nervous organ to make its appearance; that non-cephalic ganglia were gradually produced, and that the cephalic ganglion was the sole seat of sensation. But the anatomy of the Actinidæ, their scattered ocelli, ganglion and fibres, bear incontrovertible testimony to the diffusion of vitality in their bodies.

The first appearance of a cephalic mass has not hithorto been detected in the form of a separate and distinct ganglion. In the Echinoidea we see, perhaps, the simplest homologue of the vertebrates—encephalon, in the form of a ganglionated cord surrounding the gullet and sending off five branches. The nervous system is adapted to the general structure of the animal, that is, it being desirable to obtain the greatest amount of nervous power with the least occupation of space the ganglionic cord *surrounds* the œsophagus, which is short. In this manner also, the ganglia are the more readily enabled to supply the ambulacral spaces. If we now suppose one of the ganglia to retain its position on one side of the œsophagus, and the remainder of the cord to be placed on the other side of the digestive tube, but continuous and in a line with the solitary ganglion, we shall have a diagrammatic representation of the higher invertebrate nervous system, and shall understand how a ganglionated circle surrounding the oral extremity of a digestive tube, has one of its ganglia the homologue of the vertebrate brain.

But not the analogue. Neither in the echinoidea nor in the more highly differentiated nervous apparatus of the acephalous molluscs do we see anything approaching in function to a cephalic ganglion.

The nervous system of the annulosa consists of the double chain of ganglia already described. The greater number of the ganglia are post-œsophageal and represent the sympathetic of the higher animals; the pre-œsophageal ganglion being situated on the superior surface of the digestive tube is the direct homologue of the vertebrate brain. The insecta, which are, as I have said, the most important members of this order, exhibit the same type of nervous structure as its simplest forms; they, however, besides the increase of the thoracic and the decrease of the abdominal in size exhibit the most rudimentary form of a cerebro-spinal axis. There is a prolongation of the substance of the cephalic ganglion backwards in the form of two cords above and in contact with the non-cephalic ganglia. Functionally these spinal fibers unite the ganglia and fibers into a composite machine capable of obeying the dictates of any one ganglion, but more especially of the cephalic. If such annulose animals as the garden worm, in whom there are no spinal fibers, be cut into several pieces, each portion will preserve the power of movement for hours, provided each segment have a perfect ganglion: we may cut the animal into as many pieces as we choose, and each portion will preserve the power of movement. If we now take an insect, such as the house fly, decapitate it, the animal will be able to perform to a limited extent the action synonomous with its name: it will walk, if placed on its back, it will regain its footing, and perform other actions presently to be considered. Now cut the body into two segments, and what is the result? The animal lies motionless and dead. The conclusion is obvious: in the worm the separate pairs of ganglia form an independent vital apparatus; in the insect they are no longer independent but through the spinal fibers—inter-dependent. Whereas the worm is *multiple* in its points of energy, the insect is *dual*, i.e., it possesses cephalic and non-cephalic apparatus, the non-cephalic having several distinct loci, stimulation of any one calling for the action of the whole non-cephalic apparatus.

The cephalic is the most important ganglion of the worm, the whole body is capable of obeying it, *but through* the non-cephalic ganglia. The impulse proceeds backwards from ganglion to ganglion, and this is exemplified by the animals mode of progression. The worm moves by approximating the second segment to the first and the third to the second, and so on: a gradual wrinkling, shortening and lateral enlargment of the anterior portion of the body is seen, the wrinkling and approximation proceed in a rhythmical order from before backwards.

The approximation being complete, extension takes place in the same order. Now, in such a high vertebrate as the serpent, which, however, as it moves by undulatory motion may be compared for one moment with the foregoing, there is no such approximation from before backwards. Without necessarily moving its head or forepart, the snake arches its posterior extremity and an undulatory progressive motion proceeds from behind forwards. The posterior extremity of the body acts immediately in obedience to the anterior, and not by successive transmission of the impulse from ganglion to ganglion.

But to revert to the insecta. It is owing to the very inter-dependence of their parts—to the oneness in life of non-cephalic ganglia that section abolishes such oneness and cessation of action ensues. But were the nervous system not a dual—were it a unit—had the spinal fibers commanded the ganglia only in obedience to the cephalic ganglion, then on severance of such ganglion all action would cease, *but it does not.* It is not a question of the action of individual parts, but the whole body walks, flies, and the limbs move in their regular order. The animal acts like a being without a cephalic ganglion.

Let us consider that reflex action takes place in obedience to a stimulus at the periphery of a sensory nerve. When a decapitated insect is on its back, what stimulus affects the extremities of the legs to make it regain its feet? Normally the ganglia which are connected with the limbs are under the control of volitional centers; the animal, if walking, stops when it pleases, so it does when decapitated; *ergo*, there must be a volitional center.

In the highest vertebrates bodily actions may be divided into reflex and non-reflex. This, then, is the highest differentiation of efferent action. Reflex movements may take place through the brain, spinal cord and sympathetic system. They govern the visceral functions, and are concerned in many so-called voluntary actions of our daily life. So greatly is this the case that the physiologist is apt to consider the reflex power a property of the non-cephalic centres, which enables them to act independently of the brain. How thoroughly erroneous this opinion is, the execution of criminals by the axe has sufficiently proved. Death almost immediately follows such decapitation, and no bodily reflex or other actions ensue. The conclusion is that these centers are directly dependent on the encephalon for the maintenance of their energy. But if the domestic fowl be suddenly beheaded it will often walk or run several paces, and always manifests a greater amount of energy than the decapitated man.

Here the conclusion is that the non-encephalic centers are more independent of the cephalic than in man.

If the frog be subjected to the same experiment it will not merely live for several hours but will strive to push away any instrument with which it is touched.

In this case also the same law is pursued. In the insecta the same phenomena are exhibited, but intensified.

The same may be said of the worm.

If we now pass through the series, from below upwards—worm, insect, frog, fowl, man—we see more plainly *how the cephalic ganglion* is gradually increased in motor and sensory power, and the non-cephalic ganglion correlatively lessened.

The nervous system follows the law of specialization.

The ganglia of the Echinoidea are reflex and sensitive and motor, without predominance of any one ganglion.

In the worm all the ganglia possess these qualities, but the cephalic ganglion to a greater extent than the remainder.

In the Insecta the non-cephalic ganglia are combined by a continuous cord into one sensitive and motor apparatus; the individual *ganglia* are reflex. The cephalic is sensitive, motor, and reflex, and possesses the first two properties to a greater extent than any other part of the body.

The same may be said of the frog, fowl, and man. There is thus a gradual separation of parts for the more perfect performance of distinct functions, which, in the lower forms, are combined in a rudimentary condition, and are effected by undifferentiated ganglia.

In the spinal cord of the insect we see a distinct addition to the nervous system of the lowest annulose animals.

Now the cords can only be added for:—

1st.—Transmission of commands from cephalic to non-cephalic centers, or

2d.—Transmission of sensation to cephalic ganglion; or,

3d.—Differentiation of the ganglionic powers of the simplest annulosa.

With regard to the first hypothesis:—

Conducting organs cannot be *added* to a ganglionic mass without special additions to that mass, for an increased conducting apparatus can exist only when there is an increased generating apparatus.

With regard to the second hypothesis, the same may be said, substituting, however, the word 'sensitive' for 'generating.'

The cords then form the physical center of a differentiation of ganglionic powers. Is such differentiation motor or sensory or reflex? To answer this we must solve another problem—Does the seat of volition correspond to that of sensation until they become differentiated?

In man the encephalon consists of the cerebral hemispheres, the sensory ganglia, the cerebellum, pons, crura, and medulla.

The highest mental acts are Volition and Ideation.

Pathology has proved that these faculties have their seat in the cerebrum.

In the frog ideation and memory are situated in the cerebrum, but not volition entirely. Volition in this animal occupies the whole spinal axis, increasing from below upwards. So that it follows the course of sensation.

It is only in the highest vertebrates that we find ganglia differentiated into sensory and volitional. We therefore conclude that among the humbler forms the sensory are volitional.

This leaves us reflex action—the ganglia are the seat of this.

Therefore, the spinal cord of the insect is to be regarded as a center of common sensation; it acts by itself without the brain Moreover, it acts as a whole; section destroys its irritability and occasions almost immediate death.

The mammalia, aves, repilia, and pisces occupy this order with reference to their brain development, and also to their intelligence.

The average sizes of their nerve fibers are as follows:—

| | | | | |
|---|---|---|---|---|
| Mammalia . . . . | $\frac{1}{1625}$ to $\frac{1}{6500}$ | of an inch in diameter. | | |
| Aves . . . . . . | $\frac{1}{2000}$ " $\frac{1}{3000}$ | " | " | |
| Reptilia (Frog) . . | $\frac{1}{1260}$ " $\frac{1}{2280}$ | " | " | |
| Pisces (Eel) . . . . . . . . | $\frac{1}{1643}$ | " | " | |

The nerve fibers in man are smallest in the brain and spinal cord, in which they measure from $\frac{1}{10000}$th to $\frac{1}{14000}$th in. in diameter; in the trunks and branches of the nerves they measure from $\frac{1}{2000}$th to $\frac{1}{3000}$th in.; so that the fineness of the nerve fiber in man is correlated to the altitude of its functions.

The difference between the nervous power of man and of the inferior animal, and the corresponding difference in the size of the fibres shows that there is a general correlation of nerve power to the fineness of the fibers.

The relative difference in size between the fibers of the nerve branches and those of the cephalic ganglion, and between those of the latter organ and of the spinal cord, decreases as we pass downwards among the members of the animal kingdom.

Among invertebrate animals the fibers are relatively fewer in number and coarser than in the vertebrates. The fibers of the cephalic ganglion, where present, are finer than those of the nerve branches. The fibers are finer absolutely in the higher than in the lower invertebrata. We can, therefore, judge of the relative powers of parts of any animal's nervous system by comparing the size of their fibers.

The general law of whose principles I have been speaking may be thus formulated.

There is throughout the members of the animal kingdom possessing a nervous system a gradual differentiation of nervous cords to separate fibers. The differentiation is both absolute and relative. Absolute as regards the relation of the fibers of an animal to those of a member of a higher or lower order, relative as regards the cephalic and non-cephalic fibers of any one animal. The differentiation is greater in the former than in the latter case.

Throughout the cold-blooded vertebrates the proportion of the nerve centers to the nerve is much less than in the warm-blooded animals; there is thus a direct proportion between the nerve totals or systems and nerve fibers.

Evolution or development then proceeds from generally sensitive and homogeneous protoplasm to more sensitive spots, which spots are concerned in the appreciation of special qualities of external objects; these become elaborated into organs of distinct special sense—ocelli and auditory vesicles.

Tracts of cells communicating with these sense organs are enabled to conduct impressions and impulses through the body substance, without an apparent structural differentiation of such cells.

The latter condition, however, shortly obtains when we see one or more ganglia sending fibers to the various body organs.

Multiplication of ganglia now occurs without the supremacy of any one ganglion.

The next step is the establishment of the latter condition.

The further development is from the cephalic ganglion, which sends backwards two communciating cords.

Elaboration of the cerebro-spinal axis now proceeds.

The qualities of the tissues of the highest vertebrates are the differentiated properties of the simple protoplasmic cell.

To know the power of a being we have only to estimate the individual functions of its differentiated parts—if any.

Where there is one ganglion it must be the set of the animal's highest powers.

Where there are ganglia in connection with sense organs, and others in connection with muscular fibers etc., we have ganglia of special sense (sight and hearing) and *others.*

The *others* must then represent the remaining powers of protoplasm—immediate tactility—motor and reflex action.

Thus far then, there is only a differentiation of special sense.

With the prolongation backwards of the cephalic ganglion the non-cephalic ganglia yield their properties of common tactility and volition to such process retaining their power of reflex action. This is the second nervous differentiation.

Such a cerebro-spinal axis makes its first appearance in the insecta.

From this point the differentiation of nerve faculties is absolute and in kind in so far as the cephalic ganglion is concerned.

This becomes elaborated into separate ganglia having distinct functions. But the spinal cord merely differentiates in degree; it receives the power of reflex action, this increases with the multiplication of the sympathetic ganglia; at the same time, it loses by degrees its faculties of common sensation and volition, which become centralized in the cephalic ganglion. But with such centralization there is a dependence of the cord on the cephalic ganglion for the maintenance of its powers.

Hence it follows that separation of such ganglion by severance from the cord increases the functional activity of the latter, the extent of such impairment of energy being exactly correlated to the cephalic assumption of the erst general nerve properties.

If we represent the spinal power of the insecta by 100, and its cephalic by 150, and supposing the frog lose three-fifths of its energy when decapitated, we may roughly demonstrate the change as follows:—

| | |
|---|---|
| Insects' normal spinal power . . . . . | 100 |
| " cephalic " . . . . . | 150 |
| | —— |
| Total cerebro-spinal power . . . . . | 250 |

Insects' spinal powers . $100-\frac{3}{5}$ (frog's loss)=60–100=40, frog's spinal power.

Frogs' cephalic ganglion = 150 (normal in insecta) + 60 (gain in frog) = 210, normal in frog.

Examples of such increasing centralization have been given.

The action of the body in the lowest animals is effected by simple continuity of its composite particles without reference to linear transmission, or any other, save the diffused and uniform. In the higher animals such action is effected by the continuity and differentiation of certain lines of cells and subservence of the rest of the body to these.

The centralization of energy is always regional at first; that is, it occupies a tract of the body substance, it is not intercellular, but, on the contrary, involves many cells. It becomes intercellular by development.

The greater importance of the cephalic ganglion that the remainder of the ganglion is always proved when decapitation impairs energy.

That it is not the sole seat of energy is always proved when after such decapitation energy is preserved.

The development of the nervous system is in two directions: first, towards the erection of a large complex cephalic ganglion and spinal cord; and secondly, the extension into the tissues of ramifications from the nervous trunks.

Lastly, the fineness of the nerve fibers is correlated to the altitude of the functions of the nerve organ in which they are situate, and to the place and intelligence of the being in the animal world.

